# Association between accelerometer-measured physical activity volume and sleep duration in older adults: a cross-sectional interpretable machine learning analysis

**DOI:** 10.3389/fpubh.2025.1635020

**Published:** 2025-08-20

**Authors:** XiaoTao Cai, Yi Xian, YuXin Zhou, TongYi Liu, Xinyue Zhang, Qing Chen

**Affiliations:** ^1^Institute of Physical Education, Sichuan University, Chengdu, China; ^2^School of Physical Education and Spout Science, Fujian Normal University, Fuzhou, China; ^3^College of Biomedical Engineering, Sichuan University, Chengdu, China; ^4^School of Physical Education, Xizang Minzu University, Xianyang, China

**Keywords:** older adults, physical activity, sleep duration, machine learning, SHAP

## Abstract

**Objective:**

This study aimed to examine the relationship between physical activity volume and sleep duration in older adults, using objective monitoring data to investigate their non-linear association and threshold effects, thereby providing references for developing exercise programs to improve sleep duration.

**Methods:**

The study used two consecutive waves of NHANES cross-sectional data (2011–2014) as the derivation cohort and NHANES 2005–2006 data as the validation cohort. Analysis of the derivation cohort included weighted univariate analysis, weighted multivariate logistic regression, and interpretable machine learning analysis. The machine learning interpretability process involved dividing a 20% internal validation test set, using the grid search method and five-fold cross-validation to construct RF, GBDT, XGBoost, and LightGBM models, as well as a two-layer stacked ensemble model for model comparison, with external validation of the optimal model’s performance. The final model was used for SHAP interpretability analysis.

**Results:**

Logistic regression results showed a positive correlation between physical activity volume and the probability of good sleep duration. Among the constructed models, GBDT performed best, with internal validation AUC = 0.859 (0.821–0.897, *p* < 0.001) and external validation AUC = 0.707 (0.690–0.730, *p* < 0.001). SHAP analysis results indicated that physical activity volume was particularly important for sleep duration, with the association direction consistent with logistic regression results, demonstrating strong robustness of the positive correlation. The association showed non-linear relationships and threshold effects: the marginal effects of physical activity volume changes were relatively low below 7,000 MIMS and above 15,000 MIMS, with 11461.51 MIMS being the key threshold point for predicting whether older adults would have good sleep duration.

**Conclusion:**

In studies targeting sleep duration improvement in older adults, physical activity may be considered as a non-invasive intervention. When designing such programs, special attention should be given to critical thresholds and zone effects of physical activity volume. We recommend that older adults maintain a daily activity level of at least 12,000 MIMS, with 15,000 MIMS representing the optimal standard. However, potential risks associated with excessive exercise should be noted.

## 1 Introduction

Sleep is one of the fundamental components of daily life and is essential for maintaining normal physiological function. Obtaining adequate sleep of sufficient quality is increasingly recognized as an important domain of health behavior, with particularly robust associations between sleep quality and health status in older adults (1). Sleep duration serves as a valid objective measure of sleep quality (2). In recent years, adequate sleep has been incorporated into national health priorities (3). Substantial evidence demonstrates that sleep duration deviating from normative ranges (either insufficient or excessive) shows significant associations with adverse health outcomes (4), highlighting the importance of monitoring sleep patterns in aging populations.

Existing studies indicate that older adults meeting physical activity requirements are more likely to obtain appropriate sleep duration (5). Moderate physical activity not only serves as an effective approach to enhance sleep quality (6, 7)and maintain sufficient sleep duration (8), but also represents a crucial lifestyle intervention for improving sleep outcomes in middle-aged and older populations (9). Notably, those engaging in leisure walking exhibit a 34% higher probability of achieving adequate sleep duration compared to non-walkers (10). However, is there a non-linear relationship between the amount of physical activity and sleep duration, and what level of physical activity is necessary to promote appropriate sleep duration in the older population? Is there a threshold range for physical activity levels? These unresolved questions demand systematic investigation.

Traditional methods of assessing sleep duration and physical activity have relied on data from sleep duration questionnaires and physical activity questionnaires collected through self-report. Compared with objective sleep monitoring and physical activity monitoring data, there are limitations in that (1) there are differences in participants’ perceptions of physical activity levels and sleep duration, and (2) subjective recall of quantitative time is susceptible to recall bias, particularly in older populations (11). For example, the correlation between self-reported MVPA time and accelerometer-measured MVPA time was only 0.4 among adults aged 65 years or older (12). Moreover, unlike exercise, which is a structured or planned form of physical activity, physical activity consists of daily activities (e.g., commuting to and from work, doing household chores, etc.), and self-reporting cannot provide the data required for a 24-h activity model (13). With advances in wearable technology, the field of PA assessment is increasingly using wearable monitors to directly measure components of PA. Wearable monitors can more accurately assess parameters that correspond to PA (14). Physical activity volume and sleep duration quantitatively assessed by wearable accelerometers have higher accuracy than self-reported assessments. Additionally, regarding analytical methods, traditional logistic regression models are limited by their linearity assumption, making it difficult to capture the nonlinear association and threshold effects between physical activity volume and sleep duration. Therefore, we employed interpretable machine learning models, which can automatically learn complex patterns from data and effectively identify nonlinear relationships and threshold effects between variables. This study aimed to extract objective metrics of physical activity volume and sleep duration from accelerometer data while exploring their relationship after adjusting for confounding variables, ultimately developing interpretable machine learning models to assess both marginal and aggregate threshold effects of physical activity changes on sleep outcomes. To inform exercise programmes targeted at improving sleep duration in older adults.

## 2 Materials and methods

### 2.1 Data sources

The dataset was obtained from the National Health and Nutrition Examination Survey (NHANES), a cross-sectional research program conducted by the Centers for Disease Control and Prevention (CDC) since 1999. NHANES has systematically monitored the health and nutritional status of the U.S. population through standardized questionnaires, physical examinations, and laboratory tests since the 1960s. For model development, we analyzed data from two consecutive NHANES survey cycles (2011–2014) that implemented Physical Activity Monitor (PAM) examinations, comprising a total of 19,931 participants with complete activity monitoring records. We focused on adults aged 60 years or older and applied the following exclusion criteria: participants younger than 60 years (*n* = 16,299), those with insufficient accelerometer data (*n* = 863), and individuals missing key covariates including household income, marital status, BMI, smoking status, and alcohol use status (*n* = 372). The final derivation cohort comprised 2,397 eligible participants aged ≥60 years.

External validation was performed using NHANES 2005–2006 data (*N* = 10,348) through temporal validation (15, 16). This validation cohort utilized a different version of the Physical Activity Monitor (PAM) for assessing physical activity compared to the development cohort. Exclusion criteria mirrored the derivation phase: participants younger than 60 years (*n* = 8,778), those with missing accelerometer data (*n* = 328), and cases with incomplete covariate data (*n* = 169). The validation cohort ultimately included 1,073 participants. To ensure computational reproducibility, we fixed the random seed at 42 during sample extraction.

The NHANES 2011–2014 dataset employed wrist-worn ActiGraph GT3X+ accelerometers to continuously monitor participants’ 24-h physical activity patterns over seven consecutive days. The devices recorded triaxial acceleration data at 80 Hz while simultaneously collecting ambient light data at 1 Hz sampling frequency. Wear time metrics included: effective awake minutes (PAXWWMD), effective sleep minutes (PAXSWMD), and undifferentiated minutes where activity state (awake/asleep) could not be determined. Since undifferentiated minutes potentially introduce measurement error, we implemented stringent quality control criteria. A valid monitoring day required ≥1,296 minutes (17) of total wear time (PAXWWMD + PAXSWMD), excluding undifferentiated minutes. Participants were included only if they provided ≥4 valid days with at least one valid weekend day.

For NHANES 2005–2006, physical activity monitoring used the hip-mounted ActiGraph AM-7164 uniaxial accelerometer worn for seven consecutive days. This earlier protocol differed fundamentally by: (1) using single-axis rather than triaxial measurement, and (2) lacking sleep monitoring capability. We maintained comparable rigor by defining valid minutes as meeting both wear status (PAXSTAT = 1) and calibration criteria (PAXCAL = 1), with identical daily wear time requirements (≥1,296 valid minutes/day). The same inclusion threshold of ≥4 valid days was applied to ensure data quality parity across survey cycles.

### 2.2 Sleep health classification

The construction of the sleep duration classification variable in the derivation cohort was first based on the calculation of the effective average daily sleep duration ( Sleepdaily ) from wrist accelerometer data, where $\sum_{d=1}^{D\_\text{valid}}\text{PAXSWMD}$ represents the total sleep wear time across valid days and $\sum_{d=1}^{D\_\text{valid}}\text{total}\_\text{wear}\_\text{time}$ denotes the total wear time in minutes over valid days. The calculation was performed as follows:


$$\text{Sleepdaily}=\frac{\sum_{d=1}^{D\_\text{valid}}\text{PAXSWMD}}{\sum_{d=1}^{D\_\text{valid}}\text{total}\_\text{wear}\_\text{time}}\times 24$$

In the external validation cohort, due to the lack of objective sleep monitoring device data, the self-reported sleep duration from questionnaires was used as the average daily sleep duration.

After calculating the effective average daily sleep duration, we referred to the sleep duration classification established by the National Sleep Foundation (18), which categorizes sleep duration for older adults aged 65 and above into a binary variable: 0 = bad (<5 or >9 h) and 1 = appropriate (5 to 9 h).

### 2.3 Physical activity volume

The average daily physical activity volume ( PAdaily ) was calculated as follows. In the derivation cohort, the data were expressed in MIMS (Monitor-Independent Movement Summary) (19) units—a novel aggregation method for raw sub-second accelerometer data, where minute-level triaxial acceleration values (x-, y-, and z-axes) are converted into MIMS units. Here, $\sum_{d=1}^{D\_\text{valid}}\text{PAXMTSD}$ represents the sum of daily triaxial MIMS values across valid days ( $D_{\text{valid}}$ ), while $\sum_{d=1}^{D\_\text{valid}}\text{total}\_\text{wear}\_\text{time}$ denotes the total wear time in minutes over valid days. The calculation formula was:


$$\text{PAdaily}=(\frac{\sum_{d=1}^{D\_\text{valid}}\text{PAXMTSD}}{\sum_{d=1}^{D\_\text{valid}}\text{total}\_\text{wear}\_\text{time}})\times 60\times 24$$

For the validation cohort, where physical activity volume lacked standardized units, daily activity was computed by first calculating the daily total activity ( ${\text{DailyTotal}}_{d}$ , *d* where subscript *d* represents day *d*). The total valid activity volume was obtained by summing daily activity across valid days: $\sum_{d=1}^{D_{\text{valid}}}{\text{DailyTotal}}_{d}.$ The average daily physical activity volume was then derived by dividing the total valid activity by the number of valid days:


$$\text{PAdaily}=\frac{\sum_{d=1}^{D_{\text{valid}}}{\text{DailyTotal}}_{d}\;}{D_{\text{valid}}}\;$$

This continuous variable represented participants’ average daily physical activity volume. To mitigate the influence of extreme values, we applied 1% winsorization to both tails of the distribution.

### 2.4 Covariates

The study incorporated the following demographic covariates: age, sex (0 = male, 1 = female), and race/ethnicity categorized as 1 = Hispanic (including Mexican American and other Hispanic), 2 = non-Hispanic White, 3 = non-Hispanic Black, and 4 = other races. BMI was classified into 1 = BMI < 25, 2 = 25 ≤ BMI < 30, and 3 = BMI ≥ 30, with measurements obtained from physical examination in the derivation cohort and calculated from self-reported height and weight in the validation cohort. Education level was categorized as 1 = less than high school, 2 = high school graduate or equivalent, and 3 = some college or college graduate. Marital status was grouped as 1 = married/living with partner and 2 = widowed/divorced/separated/never married. Household income was stratified by poverty-to-income ratio (1 = PIR ≤ 1, 2 = 1 < PIR < 4, 3 = PIR ≥ 4) (20). Smoking status was defined as 1 = never smoker (<100 cigarettes lifetime), 2 = former smoker (≥100 cigarettes but quit), and 3 = current smoker. Alcohol drinkers (0 = non-drinker, 1 = drinker) were determined based on affirmative responses to the question: “During any 1 year, have you had at least 12 drinks of any type of alcoholic beverage?" (21).

### 2.5 Statistical methods

All data analyses were performed using SPSS and Python software. Following NHANES recommendations, we incorporated the Mobile Examination Center (MEC) examination weights (WTMEC2YR) in all analyses, as some study variables were collected during MEC examinations. Participant characteristics were described using weighted means (standard deviation, SD) and frequencies (weighted percentages). Group differences were assessed using weighted t-tests for continuous variables and weighted chi-square tests for categorical variables.

In the derivation cohort, weighted multivariable logistic regression was initially employed to explore the association between physical activity volume and sleep duration, with adjustments made for sociodemographic, behavioral, and other confounding factors. Two adjusted models were developed to verify result robustness. Variables showing *p* < 0.05 in univariate analyses were subsequently incorporated into four machine learning algorithms: Random Forest (RF), Gradient Boosting Decision Tree (GBDT), eXtreme Gradient Boosting (XGBoost), and Light Gradient Boosting Machine (LightGBM). Optimal hyperparameters were determined through GridSearchCV with 5-fold cross-validation, and models were internally validated using test datasets.

Model selection was based on comparison of the area under the receiver operating characteristic curve (AUC) in test datasets, with additional evaluation of predictive performance using accuracy, precision, recall, and F1-score metrics (22). The final model underwent external validation using the independent validation cohort. All tests were two-sided with significance level *α* = 0.05.

For model interpretation, we applied SHAP (SHapley Additive exPlanations) values, an algorithm developed by Lundberg and Lee based on Shapley values from cooperative game theory (23). This approach quantifies each variable’s contribution to model predictions, providing additive feature importance measures for interpreting the “black-box” machine learning model.

**Figure 1:**
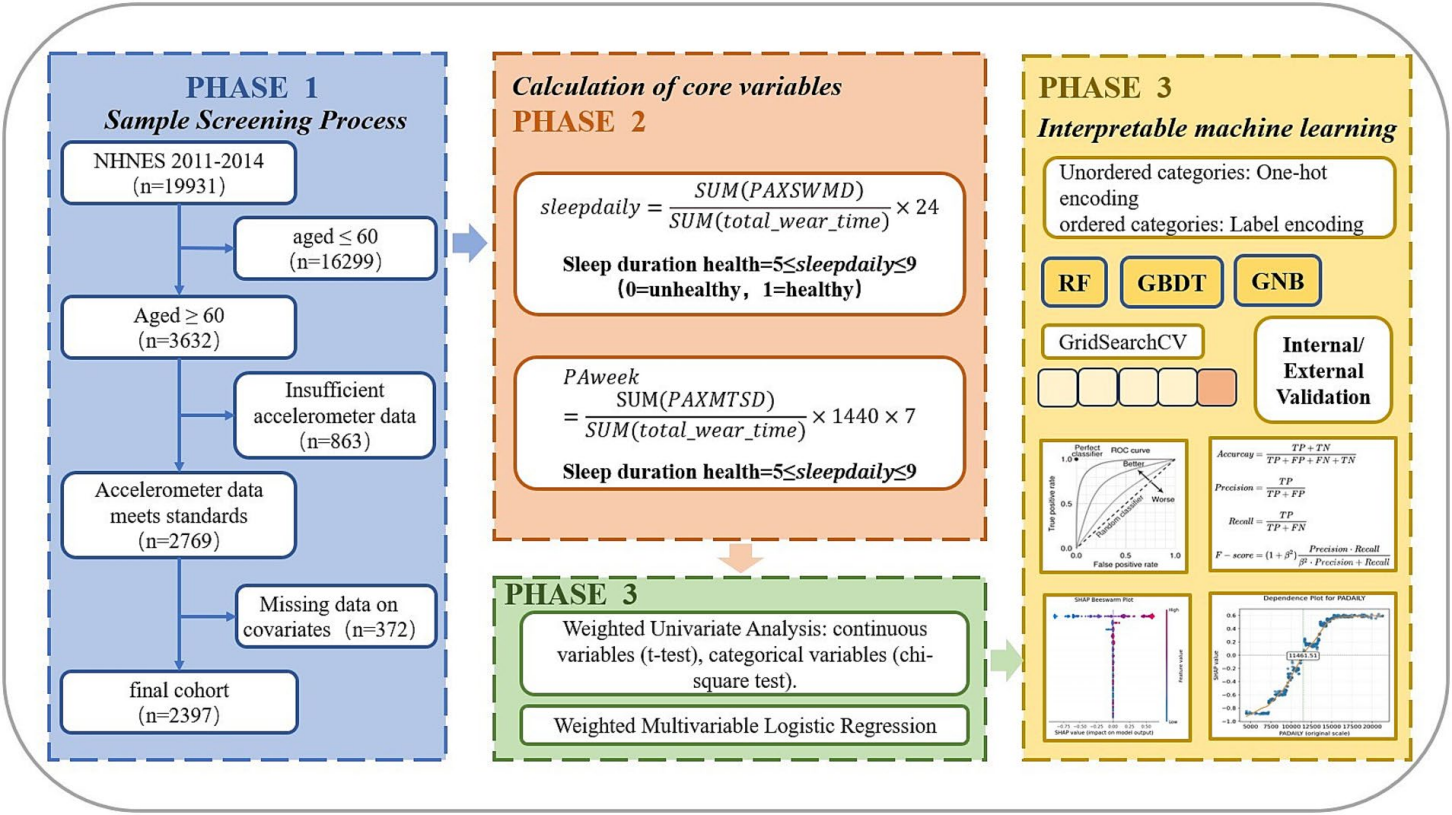
Research flowchart.

In summary, the study flowchart is presented in Figure 1.

## 3 Results and analysis

### 3.1 Comparison of baseline characteristics between groups

The distribution of the primary study factor, sleep duration, among the two groups of older adults is presented in Table 1. Statistically significant differences (*p* < 0.05) were observed between the groups for the following variables: age (*p* < 0.001), sex (*p* < 0.001), race/ethnicity (*p* < 0.001), BMI (*p* < 0.001), education level (*p* < 0.001), marital status (*p* < 0.001), household income (*p* < 0.001), smoking status (*p* < 0.001), alcohol consumption (*p* < 0.001), and total daily physical activity volume (*p* < 0.001). The comparison of baseline characteristics between groups in the external validation cohort is provided in Supplementary Table 1.

**Table 1:** Baseline characteristics of participants stratified by sleep duration (≥60 Years): NHANES 2011–2014.

Variable	Subgroup	Bad sleep duration (*N* = 37′097’763, 42.9%)	Appropriate sleep duration (*N* = 49′311’496, 57.1%)	*p*-value
Age		71.30 ± 6.85	68.20 ± 6.29	<0.001
Sex (%)				<0.001
	Male	45.6	54.4	
	Female	40.8	59.2	
Race/ethnicity (%)				<0.001
	Hispanic	32	68	
	Non-Hispanic white	45.5	54.5	
	Non-Hispanic Black	34.4	65.6	
	Other races	32.6	67.4	
BMI (%)				<0.001
	<25	36.5	63.5	
	[25, 30)	43.9	56.1	
	≥30	46.3	53.7	
Education (%)				<0.001
	Elow high school	45.7	54.3	
	High school	49.1	50.9	
	College or above	40	60	
Marital status (%)				<0.001
	Married/living with partner	41.3	58.7	
	Widowed/divorced/separated/never married	45.8	54.2	
Household income (%)				<0.001
	PIR ≤ 1	44.2	55.8	
	1 < PIR < 4	46.9	53.1	
	PIR ≥ 4	36.6	63.4	
Smoking status (%)				<0.001
	Never smoker	41.1	58.9	
	Former smoker	46.5	53.5	
	Current smoker	38	62	
Alcohol drinkers (%)				<0.001
	Non-drinker	40.3	59.7	
	Drinker	44.0	56.0	
Physical activity volume		9728.54 ± 2686.34	13693.94 ± 2919.19	<0.001

### 3.2 Weighted logistic regression analysis

Table 2 presents the odds ratios (ORs) and 95% confidence intervals (CIs) for the association between total physical activity volume and sleep duration. The crude analysis showed an OR of 1.000525 (95% CI: 1.000524–1.000525). After adjustment for age and race/ethnicity, the OR for appropriate sleep duration was 1.000539 (95% CI: 1.000539–1.000539). Further adjustment for additional covariates including BMI, household income, smoking status, and alcohol consumption yielded an OR of 1.000563 (95% CI: 1.000563–1.000564), with all models showing statistical significance (*p* < 0.001). Furthermore, interpreting the odds ratio in the context of physical activity measurement scale is essential. To evaluate the clinical relevance of our findings, we employed the minimal clinically important difference (MCID) approach. While MCID can be determined through various methods including criterion, distribution-based, literature analysis, and expert consensus approaches, the limited existing research on MIMS units constrained the applicability of literature analysis and expert consensus methods. The criterion approach would also require additional clinical validation. Therefore, we adopted the distribution-based method for MCID estimation. Refer to existing research (24), we defined MCID as 0.5 times the baseline standard deviation (SD). The analysis revealed an SD of 3437.27 for physical activity levels, yielding an MCID of 1718.64 MIMS (0.5 × SD). This indicates that for every MCID-unit (1718.64 MIMS) increase in physical activity, the odds of achieving appropriate sleep duration improve by 2.6309-fold compared to the reference group with poorer sleep duration. The calculation formula is as follows.


$$OR_{\text{MCID}}={\left(1.000563\right)}^{1718.64}=2.6309.$$

**Table 2:** Odds ratios (95% CI) of sleep duration by physical activity levels.

Variable	Crude model ^a^	Model 1 ^b^	Model 2 ^c^
PAdaily	1.000525 (1.000524,1.000525)	1.000539 (1.000539,1.000539)	1.000563 (1.000563,1.000564)

OR, odds ratio; CI, confidence intervals.

a Crude model: no covariates were adjusted.

b Model 1: age, sex, and race/ethnicity were adjusted.

c Model 2: age, sex, race, BMI, marital status, education, household income, smoking status, and alcohol drinking status were adjusted.

Finally, to ensure the robustness of our findings, we conducted sensitivity analyses by redefining appropriate sleep duration as 7–8 h (representing 20.8% of the total sample) as the positive outcome. After performing weighted multivariate logistic regression using the same analytical pipeline, the sensitivity results remained consistent with our primary findings, demonstrating a positive association between physical activity volume and the probability of appropriate sleep duration (OR = 1.000207, *p* < 0.001).

These results demonstrate that higher physical activity volume remained significantly associated with increased probability of appropriate sleep duration after comprehensive adjustment for potential confounding factors.

### 3.3 Model development and hyperparameter optimization

The predictive model for sleep duration classification was constructed using 10 variables identified as significant (*p* < 0.05) in univariate analyses as input features, with continued application of WTMEC2YR weighting. To enhance model interpretability and avoid imposing spurious ordinal relationships, categorical variables were processed as follows: nominal features underwent one-hot encoding while ordinal features received label encoding. The dataset was partitioned into training (80%) and independent test (20%) sets while preserving the weighted survey design.

For hyperparameter optimization, we implemented an exhaustive grid search strategy (GridSearchCV) with 5-fold cross-validation across four machine learning algorithms: Random Forest (RF), Gradient Boosting Decision Tree (GBDT), eXtreme Gradient Boosting (XGBoost), and Light Gradient Boosting Machine (LightGBM). This systematic approach evaluated all possible parameter combinations within predefined search spaces to identify globally optimal configurations, thereby maximizing model generalizability and computational efficiency. Key parameters were optimized as shown in Table 3, while non-critical parameters retained their default values.

**Table 3:** Optimal hyperparameter combinations for each algorithm.

Model	Hyperparameter	Optimal value
RF	bootstrap	True
	max_depth	5
	max_features	“sqrt”
	min_samples_leaf	1
	min_samples_split	2
	n_estimators	100
GBDT	learning_rate	0.01
	max_depth	3
	min_samples_split	2
	n_estimators	50
	subsample	0.8
XGBoost	colsample_bytree	0.8
	learning_rate	0.01
	max_depth	3
	n_estimators	50
	subsample	0.8
LightGBM	learning_rate	0.01
	max_depth	-1
	min_child_samples	20
	n_estimators	100
	num_leaves	31

### 3.4 Model performance comparison

As shown in Table 4, bootstrap resampling (*n* = 100 iterations) was employed for model performance evaluation. The results demonstrated that all four models achieved AUC values exceeding 0.80 on the test set: RF (AUC = 0.851, 95% CI: 0.800–0.896, *p* < 0.001), GBDT (AUC = 0.859, 95% CI: 0.821–0.897, *p* < 0.001), XGBoost (AUC = 0.852, 95% CI: 0.813–0.893, *p* < 0.001), and LightGBM (AUC = 0.847, 95% CI: 0.807–0.883, *p* < 0.001). These results indicate excellent discriminative ability in predicting sleep duration among older adults, with the GBDT model showing superior overall predictive performance compared to other individual models.

**Table 4:** Test set model results.

Model	AUC	AUC 95%CI	Accuracy	Precision	Recall	F1-score
RF	0.851	(0.800–0.896)	0.796	0.774	0.882	0.824
GBDT	0.859	(0.821–0.897)	0.769	0.725	0.928	0.813
XGBoost	0.852	(0.813–0.893)	0.757	0.710	0.937	0.807
LightGBM	0.847	(0.807–0.883)	0.759	0.739	0.861	0.795
Stacking	0.837	(0.790–0.871)	0.742	0.722	0.856	0.783

To further enhance generalization capability, we developed a two-level stacked ensemble architecture using GBDT as the meta-learner and RF, XGBoost, and LightGBM as base-learners. The ensemble model achieved an AUC of 0.837 (95% CI: 0.790–0.871, *p* < 0.001), which was slightly lower than the standalone GBDT model (ΔAUC = 0.022). This may be attributed to the similarity among the tree models used for integration, but it further confirms the optimal generalization capability of traditional GBDT models in our specific research context.

**Figure 2:**
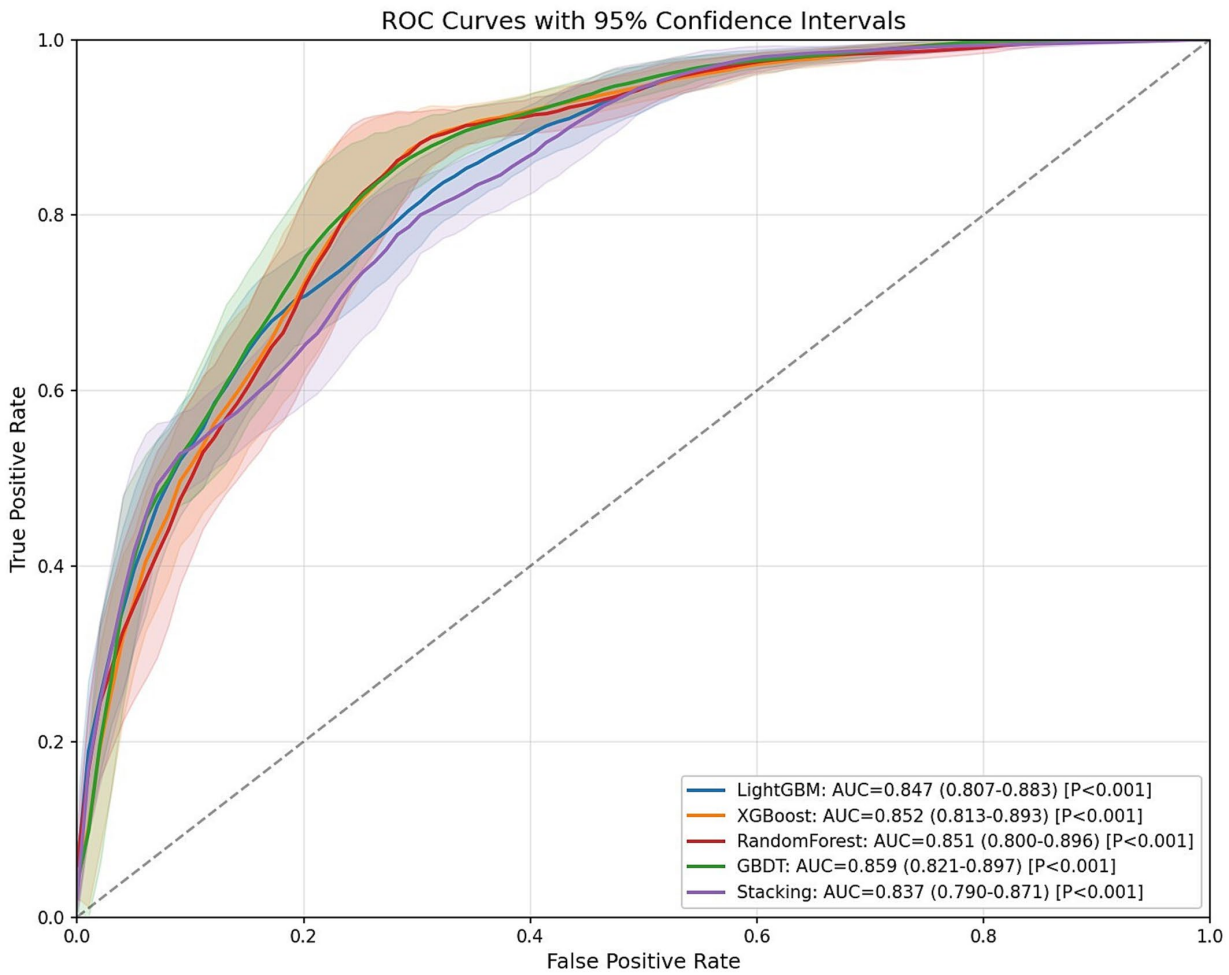
ROC curve of the machine learning model on the test set (95% CI).

Figure 2 displays the receiver operating characteristic (ROC) curves for the four prediction models. The x-axis represents the false positive rate (FPR, 1-specificity), while the y-axis indicates the true positive rate (TPR, sensitivity). In ROC analysis, points closer to the upper-left corner correspond to higher model accuracy. The area under the ROC curve (AUC) serves as a comprehensive performance metric, where larger AUC values indicate superior predictive accuracy.

### 3.5 Model robustness analysis

To further assess model stability and technical robustness while minimizing generalization risks, we performed systematic sensitivity analysis on six critical hyperparameters of the optimal GBDT model. Centered on the grid-search-optimized parameters, we evaluated one incremental step above and below each parameter value, generating 729 distinct parameter combinations as specified in Table 5. The analysis revealed remarkable parameter stability, with the model achieving a mean AUC of 0.8641 and a standard deviation of merely 0.0023, corresponding to a negligible coefficient of variation of 0.26%. The AUC values remained consistently high across all tested configurations, ranging from 0.8585 to 0.8678 with a minimal total variation of 0.93%. These findings demonstrate that the model’s predictive performance is highly resilient to hyperparameter adjustments, thereby confirming the reliability of our research conclusions against potential parameter fluctuations.

**Table 5:** Sensitivity analysis hyperparameter range.

Hyperparameters	Test scope	Benchmark optimal value	Quantity
learning_rate	[0.005, 0.01, 0.015]	0.01	3
max_depth	[3, 4, 5]	4	3
min_samples_leaf	[4, 5, 6]	5	3
min_samples_split	[10, 12, 14]	12	3
n_estimators	[150, 160, 170]	160	3
subsample	[0.75, 0.8, 0.85]	0.8	3

### 3.6 External validation

**Figure 3:**
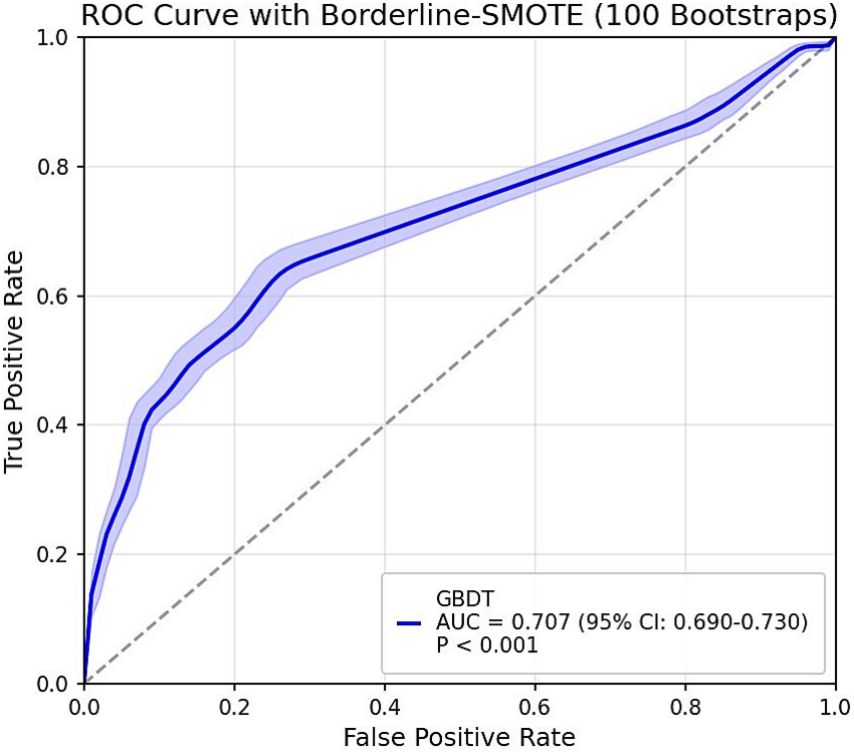
Final model external validation ROC curve (95% CI).

The GBDT model developed in the derivation cohort was applied to predict outcomes in a weighted external validation set. The validation set revealed significant class imbalance between adequate sleep duration (92%) and poor sleep duration (8%). To address this imbalance, we implemented the improved SMOTE algorithm Borderline-SMOTE (25). For sensitivity analysis, we employed the hybrid SMOTE-ENN (26) technique (combining oversampling and undersampling) rather than single undersampling methods to preserve critical information while enhancing sample purity. All sampling procedures used a fixed random seed of 42 to ensure reproducibility. The final ROC curves are presented in Figure 3. Although differences in physical activity measurement units and sleep duration assessment methods between the derivation and validation cohorts might theoretically compromise predictive performance, the validation results demonstrated robust performance: AUC = 0.707 (95% CI: 0.690–0.730) with Borderline-SMOTE balancing and AUC = 0.754 (95% CI: 0.716–0.792) with SMOTE-ENN balancing, indicating stable predictive outcomes. However, the observed reduction in generalizability compared to internal validation may be attributable to: (1) different accelerometer models with incompatible measurement units between cohorts, and (2) potential reporting bias in the externally-collected self-reported sleep data, which might lead participants to report more socially desirable sleep durations.

### 3.7 SHAP interpretability analysis

**Figure 4:**
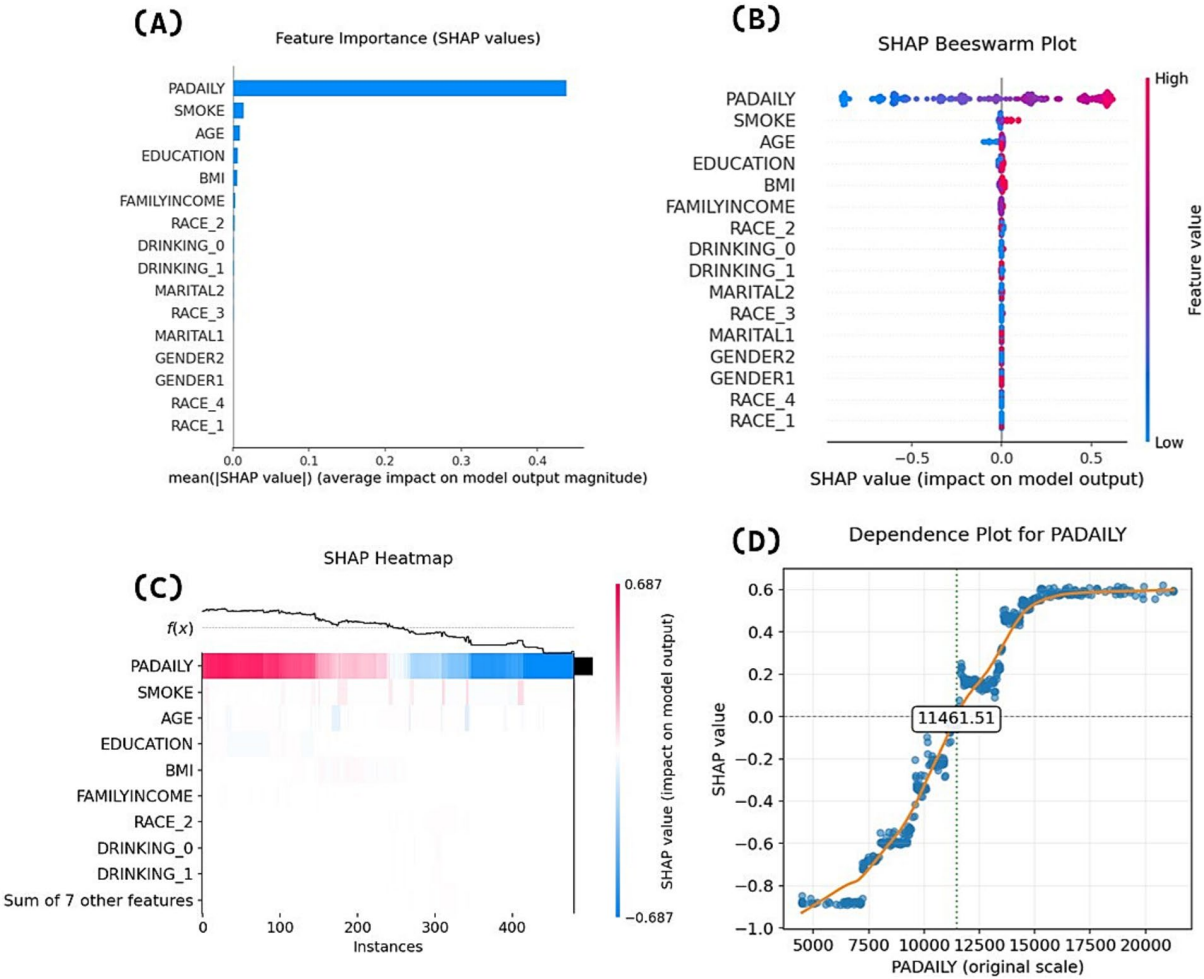
SHAP interpretation analysis panel.

Following model development, we conducted SHAP interpretability analysis on the final GBDT model. Figure 4A ranks predictive variables by mean absolute SHAP values, demonstrating that physical activity volume exerts the most significant influence on appropriate sleep duration. Figure 4B’s beeswarm plot displays individual samples (y-axis) and their impact on predictions (x-axis), where the color gradient (red = high values, blue = low values) reveals a positive association between higher physical activity levels and increased probability of appropriate sleep duration—consistent with logistic regression results. Figure 4C’s heatmap visualizes SHAP values for each participant’s physical activity volume: red/blue regions, respectively, indicate increased/decreased probability of appropriate sleep duration prediction, while the f(x) curve demonstrates a clear declining trend in overall probability as physical activity decreases (color gradient transitioning from red to blue).

The analysis of variable importance and directionality in Figures 4A–C demonstrates the association between physical activity volume and sleep duration in terms of magnitude and direction, but cannot explain potential threshold effects or nonlinear relationships across different physical activity volume ranges. Therefore, this study further generated SHAP feature dependency plots to visualize the dynamic response of model outputs (predicted values) to varying physical activity volume, as shown in Figure 4D. The horizontal axis represents physical activity volume values (MIMS), while the vertical axis indicates SHAP values. When SHAP values exceed zero, the model demonstrates a stronger propensity to predict positive class outcomes (appropriate sleep duration). The dependency plot reveals distinct threshold effects in the physical activity volume-sleep duration relationship. When daily physical activity volume was below 7,000 MIMS, the model tended to predict bad sleep duration with relatively small effects of physical activity volume changes on SHAP values, indicating limited marginal effects. In the 7,000 − 11461.51 MIMS range, while the model still predominantly predicted bad sleep duration, the slope of SHAP value changes increased significantly, demonstrating that physical activity volume increases could more effectively improve the probability of appropriate sleep duration during this phase. When daily physical activity volume exceeded 11461.51 MIMS, the model was more likely to predict appropriate sleep duration in older adults. As physical activity volume further increased beyond 15,000 MIMS, subsequent SHAP value changes stabilized, suggesting gradually diminishing marginal effects of additional physical activity volume.

## 4 Causal forest analysis

**Figure 5:**
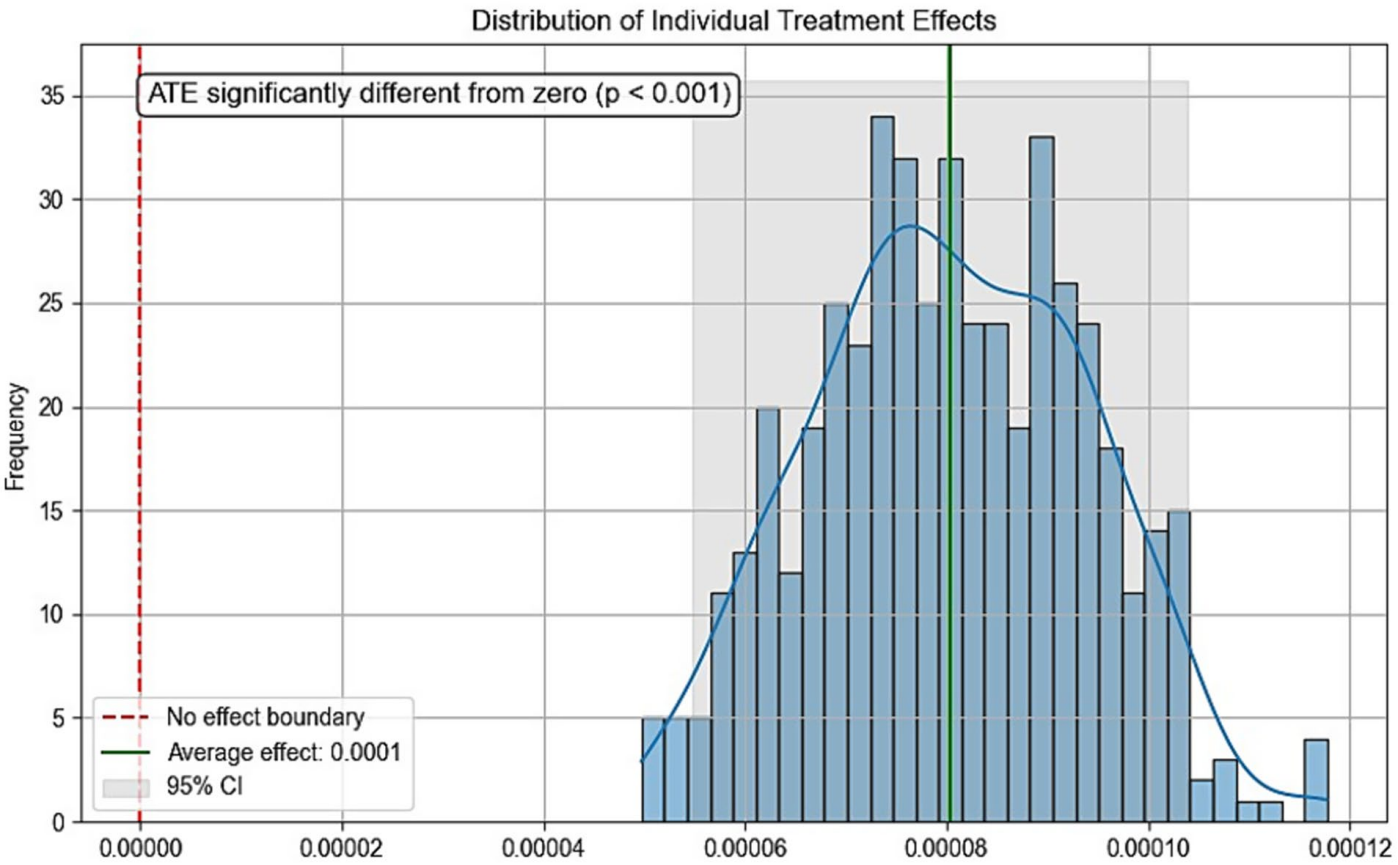
Conditional local average treatment effects.

Due to the inherent limitations of cross-sectional studies, definitive causal relationships cannot be established. To further evaluate the validity of the dose–response analysis, we employed causal forest—a tree-based causal machine learning method that demonstrates high performance in analyzing causal effects of various interventions on outcomes (27). Physical activity volume served as the treatment variable, sleep duration as the outcome variable, with additional covariates included. Parameters were set as: n_estimators = 1,000, max_depth = None, min_samples_split = 10, random_state = 42. Figure 5 displays the conditional local average treatment effects (CLATE), showing 100% of samples exhibited positive treatment effects: ATE > 0 (*p* < 0.001) with all ITE > 0. These results support the causal hypothesis that increased physical activity benefits sleep duration, thereby strengthening the theoretical rationale of SHAP analysis in interpreting dose–response relationships.

## 5 Conclusion

This study was the first to explore the relationship between physical activity volume and sleep duration in older adults through objective monitoring data and using the SHAP interpretation of the GBDT model, with excellent generalisation ability AUC = 0.859 (0.821–0.897, *p* < 0.001) for the internal validation of the model, and continued to maintain good predictive performance on the external validation set AUC = 0.707 (0.690–0.730, *p* < 0.001).

The SHAP bar plot results indicate that physical activity volume holds relatively high SHAP importance in older adults’ sleep duration. Existing studies suggest that non-pharmacological interventions should be the first-line treatment for sleep problems (28), and physical activity as a non-invasive approach to improve sleep duration in older adults warrants consideration. Multiple logistic regression, SHAP beeswarm plots, and SHAP heatmaps collectively demonstrated a positive correlation between physical activity volume and the probability of appropriate sleep duration, indicating robust findings.

The feature dependence analysis revealed a non-linear relationship with threshold effects between physical activity volume and sleep duration status, with the SHAP model identifying a critical threshold of 11461.51 MIMS. To enhance the clinical applicability of these findings, we performed rounding optimization based on the minimal clinically important difference (MCID = 1,718.64 MIMS): the range of ±0.5MCID (10,602–12,321 MIMS) was considered an equivalent interval. Given the positive association between these parameters, the highest thousand-digit integer value within this range (12,000 MIMS) was selected as the final threshold, with daily physical activity levels above this value demonstrating higher probability of achieving appropriate sleep duration.

The association between physical activity and sleep duration status can be divided into low-efficiency zone (<7,000 MIMS): the marginal effect of physical activity volume on sleep improvement is weak, and an expert consensus suggests that hypothesized reasons for non-response to exercise in health outcomes among older adults include insufficient stimulus (29), therefore lower total physical activity volume may not effectively trigger mechanisms regulating the association between physical activity volume and sleep. Sensitive growth zone (7,000–15,000 MIMS): increasing activity volume at this stage can significantly improve the probability of appropriate sleep duration. When physical activity volume reaches 12,000 MIMS or above, the probability of appropriate sleep duration will be higher than that of poor sleep duration. Saturation zone (>15,000 MIMS): excessive activity at this point will produce diminishing marginal returns and carries risks of overexercise, which may cause significant physical harm (30). Therefore, as a painless approach, physical activity should be included in prescriptions for improving sleep duration in older adults. The identified zones and thresholds should be noted. It is recommended that older adults achieve a daily average activity level of at least 12,000 MIMS, with 15,000 MIMS being optimal. These results can serve as a reference for subsequent research, but due to the current limited studies on MIMS unit cut-off values, they cannot be simply regarded as clinical recommendations.

However, this study has several limitations. First, as a cross-sectional design, it cannot establish definitive causal relationships and may be subject to potential bidirectional causality—for instance, poor sleep quality might conversely lead to reduced physical activity. Although we conducted causal forest analysis to support the causal hypothesis, this approach cannot conclusively confirm the unidirectional causal effect of physical activity on sleep duration or completely rule out the possibility of reverse causation. Second, the measurement of total physical activity volume and sleep patterns may not completely capture the dynamic patterns of physical activity. For example, two individuals may have the same total physical activity volume or sleep duration, but achieve these amounts through completely different activity patterns or sleep rhythms. Third, although accelerometer measurements have advantages over self-reported measurements, they are still affected by inter-device differences, measurement errors, and detection limits. Third, due to the different models of wearable devices used in the two selected cohorts (which also have different physical activity units) and the lack of objectively collected sleep data in the form of self-reported sleep duration, there may be a decrease in predictive ability during external validation. Fourth, MIMS units are a new metric, although research shows MIMS has better error rates and generalizability than other wearable device units (31). However, there are currently no thresholds available for classifying MIMS or determining compliance with physical activity guidelines. Therefore, interpreting the relevance of these results currently presents some difficulties. Although we used MCID for analysis, the distribution-based MCID measurement relies on statistical significance and has limitations. To summarize, future research could further explore more physical activity characteristics and clarify MIMS-based exercise intensity classifications by collecting longitudinal wearable device data, selecting more diverse external validation methods, and applying signal processing techniques.

## Data Availability

The datasets presented in this study can be found in online repositories. The names of the repository/repositories and accession number(s) can be found below: https://www.cdc.gov/nchs/nhanes/.
