# Supplementary material for: Association between accelerometer-measured physical activity volume and sleep duration in older adults: a cross-sectional interpretable machine learning analysis
Source: Front Public Health. 2025 Aug 20;13:1635020. doi: 10.3389/fpubh.2025.1635020 (PMC12404940; doi:10.3389/fpubh.2025.1635020)
Supplement: Supplementary file 1 [file Table_1.DOCX]

Supplementary Material

# Supplementary Data

Table 1 Baseline Characteristics of Participants Stratified by Sleep Duration (≥60 Years): NHANES 2005-2006

| Variable | Subgroup | Bad sleep duration  (N=2'816'038，8%) | Appropriate sleep duration  (N=32'496'012，92%) | P-value |
| --- | --- | --- | --- | --- |
| Age |  | 74.07±7.809 | 70.21±7.477 | <0.001 |
| Sex (%) |  |  |  | <0.001 |
|  |  | 6.6 | 93.4 |  |
|  |  | 9.0 | 91.0 |  |
| Race/ethnicity (%) |  |  |  | <0.001 |
|  | Hispanic | 9.1 | 90.9 |  |
|  | Non-Hispanic white | 7.2 | 92.8 |  |
|  | Non-Hispanic Black | 13.1 | 86.9 |  |
|  | Other races | 14.9 | 85.1 |  |
| BMI (%) |  |  |  | <0.001 |
|  | <25 | 7.9 | 92.1 |  |
|  | [25, 30) | 7.7 | 92.3 |  |
|  | ≥30 | 8.3 | 91.7 |  |
| Education (%) |  |  |  | <0.001 |
|  | Elow high school | 15.3 | 84.7 |  |
|  | High school | 6.2 | 93.8 |  |
|  | College or above | 5.1 | 94.9 |  |
| Marital status (%) |  |  |  | <0.001 |
|  | Married/Living with partner | 4.9 | 95.1 |  |
|  | Widowed/Divorced/Separated/Never married | 13.6 | 86.4 |  |
| Household income (%) |  |  |  | <0.001 |
|  | PIR≤1, | 16.5 | 83.5 |  |
|  | 1<PIR<4 | 8.3 | 91.7 |  |
|  | PIR≥4 | 4.5 | 95.5 |  |
| Smoking status(%) |  |  |  | <0.001 |
|  | Never smoker | 8.4 | 91.6 |  |
|  | Former smoker | 7.0 | 93.0 |  |
|  | Current smoker | 9.9 | 90.1 |  |
| Alcohol drinkers (%) |  |  |  | <0.001 |
|  | Non-drinker | 11.1 | 88.9 |  |
|  | drinker | 6.4 | 93.6 |  |
| physical activity volume |  | 119432.98±86831.10 | 173831.81±106387.12 | <0.001 |
